# Removal of arsenic with functionalized multi-walled carbon nanotubes (MWCNTs-COOH) using the magnetic method (Fe_3_O_4_) from aqueous solutions

**DOI:** 10.1039/d3ra04803a

**Published:** 2023-08-23

**Authors:** Masoumeh Khorasani Alamdari, Ata Allah Nadiri, Hossein Ghaforian, Sina Sadeghfam

**Affiliations:** a Department of Marine Science and Technology, Islamic Azad University North Tehran Branch Tehran Iran khorasanimasoume@yahoo.com; b Department of Natural Sciences, Tabriz University Tabriz Iran Nadiri.ata@gmail.com; c Marine Science and Technology Department, Islamic Azad University North Tehran Branch Tehran Iran Ghaforian25@yahoo.com; d Department of civil Engineering, Faculty of Engineering, University of Maragheh Iran s.sadeghfam@gmail.com

## Abstract

Heavy metals such as arsenic are one of the most important water pollutants and cause many environmental problems. One of the mechanisms for removing arsenic from aqueous media is the adsorption process. In this study, we investigated the efficiency of magnetized multi-walled carbon nanotubes with iron oxide (Fe_3_O_4_) nanoparticles. The precipitation method was used to synthesize Fe_3_O_4_ on PAC-(Fe_3_O_4_-f/MWCNTs) functionalized multi-walled carbon nanotubes. The effects of pH, contact time, amount of adsorbent, and contaminant concentration on the adsorption process were examined. Residual arsenic concentration was measured using induction chromatography and inductively coupled plasma mass spectrometry (ICP-MS). The physical and structural characteristics of the adsorbent were analyzed using XRD, TEM, FT-IR, TGA-DTA, BET, FESEM-EDS, Raman spectrum and X-ray. Optimal conditions for arsenic removal were pH = 2, As concentration = 6 mg L^−1^, and contact time = 30 minutes, using 0.02 g of adsorbent at room temperature. Also, fitting regression curves to the results showed that the Freundlich model (*R*^2^ > 0.9981) and a pseudo-second-order model (*R*^2^ = 1) best describe the isothermal and kinetic models of the adsorption process, respectively.

## Abstract 2

Here we show superparamagnetic iron oxide nanoparticles coated carbon nanotubes (CNTs) are easily synthesized, highly stable, biocompatible, with outstanding magnetic features. The effect of pH, contact time, contaminant concentration, and adsorbent amount on the adsorption process was measured.

## Graphical abstract

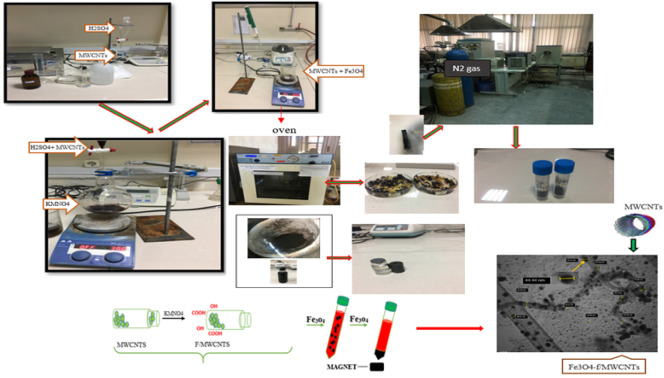

## 1. Introduction

Arsenic is widely found in the environment. On average, the Earth's crust contains 1.8 ppm of arsenic, with its concentration in soil ranging from 5.5 to 31 ppm. This element is naturally found in sulfide minerals such as orpiment and realgar, and is released during weathering and soil erosion. In recent decades, activities such as silver, gold, and gemstone mining and manufacturing of iron hydroxide magnets, TV screen, and cathode-ray tubes have released As into water. Additionally, some pesticides (including insecticides and herbicides) contain arsenic, and their uncontrolled use releases this element into agricultural runoff, increasing the arsenic concentration in soil, and subsequently in human food. When arsenic is introduced into water, it finds its way first to surface water, followed by groundwater, which can then enter the human body through drinking water.^1^ According to the World Health Organization (WHO) the maximum allowable concentration of As is 10 mg L^−1^.^2,3^ At high concentrations, As can cause nervous, gastrointestinal, and skin disorders and lead to toxicity if present in livestock feed.^4,5^

Several methods are used to remove dangerous pollutants from different media including ozonation, irradiation with UV light, use of hydrogen peroxide and the Fenton reaction, and adsorption. Among these, surface adsorption solutions are preferred.^6^

Multi-walled carbon nanotubes (MWCNTs) can be used to adsorb organic and inorganic compounds from water due to their large specific surface area, high permeability, layered hollow structure, and high adsorption potential.^7–9^ Despite their potential in water treatment, MWCNTs face challenges such as causing secondary pollution and the difficulty of removing these particles from the medium. However, by magnetizing the adsorbent, MWCNTs can be removed from treated water using an external magnet. Moreover, the presence of iron oxide compounds in Fe_3_O_4_/functional multi-walled carbon nanotubes (Fe_3_O_4_-f/MWCNTs) increases chemical stability and recoverability while reducing toxicity.^10,11^

Researchers such as Chirini *et al.*, 2008 report that surface adsorption is one of the better physical techniques for water treatment due to lower costs, ease of design, better access, and the ability to remove heavy metals. In this study, chemical activators including sulfuric acid and sodium permanganate were used to optimize MWCNTs, MWCNTs were then magnetized through iron deposition. Finally, As was removed from an aqueous environment using the fabricated MWCNTs.^12^

Oliveira *et al.*, (2021) overall in this work, a chemical noncovalent method (addition of surfactants) combined with mechanical energy (ultrasounds) was applied for CNTs stabilization, and the influence in heavy metal ions removal, Pb(ii), Cu(II), Ni(ii) and Zn(ii), an area of high environmental relevance, was evaluated. It was proven that high amounts of metals could be removed from water during the first eighteen hours. Finally, the results obtained show that MWCNTs, if adequately dispersed, present a good solution for the treatment of water contaminated with highly toxic heavy metals, even when using very low concentrations of Multiwall Carbon Nanotubes (MWCNTs). Pluronic F-127 combined with MWCNTs was revealed to be the best combination for cations removal, removing 99% of Pb(ii), 94% of Cu(ii), 90% of Zn(ii) and 76% of Ni(ii) from the aqueous systems. In general terms, the better efficiency on cations removal achieved when Pluronic F-127 was used as surfactant can be related to the smaller size of this molecule, which, while allowing a good dispersion of the MWCNTs, leaves more free active sites for the heavy metal cations adsorption on the carbon nanotubes surface. Finally, if a good dispersion of the MWCNTs is guaranteed, this nanomaterial proved to be an efficient solution for the removal of heavy metals from contaminated waters, even when using a very low concentration of MWCNTs (0.01% w/w)^13^ Wang *et al.*, (2021). In Wang *et al.*, 2021 a new type of recyclable adsorbent is synthesized through the oxidation of enhancer and modification with magnetic nanoparticles. The new adsorbent not only inherits the advantages of multiwall carbon nanotubes (6O-MWCNTs), but also exhibits a new magnetic property and further improved adsorption capacity, which is conducive to the magnetic separation and recovery of heavy metals. Te adsorption experiment shows that 6O-MWCNTs@Fe_3_O_4_ have good selective adsorption performance for Pb(ii), with a maximum adsorption capacity of 215.05 mg g^−1^, which is much higher than the existing adsorption capacity of the same type of adsorbents. And electrostatic attraction, the C=O bond in the –COOH group is induced to open by the metal ions and transforms into an ionic bond, and the metal ions are stably adsorbed on the surface of 6O-MWCNTs@Fe_3_O_4_.^14^ Kosa *et al.*, (2012) multi-walled carbon nanotubes (MWCNTs) were modified with 8-hydroxyquinoline and used for the removal of Cu(ii), Pb(ii), Cd(ii) and Zn(ii) from aqueous solutions. Fourier transform infrared spectroscopy, and X-ray photoelectron spectroscopy showed the successful modification of the MWCNTs with 8-hydroxyquinoline. The results showed that most of the metals were removed from aqueous solution using 250 mg of MWCNTs at pH 7.0 and 298 K in 0.01 M KNO_3_ after 10 min of adsorption. The results also showed that the competition between the target heavy metals was in the order of Cu(ii) > Pb(ii) ≈ Zn(ii) > Cd(ii) for % adsorption. The recycling, desorption and regeneration of the MWCNTs were evaluated and the results demonstrated that most of the metal ions desorbed at pH values lower than 2.0, and the MWCNTs could be used for up to three cycles of adsorption/desorption without losing efficiency.^15^ Ghani, *et al.*, (2021) future research works on developing a cost-effective way of nanocomposite production and toxicity testing of nanomaterials in wastewater applications are recommended. Further studies on the efficiency of the nanoadsorbents in pilot or industrial scale are highly needed to test the practicality of the nanoadsorbents for selected heavy metals removal from real wastewater. To date, this work presented a brief review of a wide range of nanoadsorbents had been successfully utilized for heavy metals adsorption from wastewater with excellence and high removal.^16^ Gusain *et al.*, (2021) in this study, we investigate the adsorption capability of molybdenum sulfide (MoS_2_)/thiol-functionalized multiwalled carbon nanotube (SH-MWCNT) nanocomposite for rapid and efficient removal of heavy metals [Pb(ii) and Cd(ii)] from industrial mine water. MoS_2_/SH-MWCNT nanocomposite was successfully prepared following a facile hydrothermal approach. Enhanced interlayer spacing of MoS_2_ nanosheets was achieved by intercalation of Na or hydrated Na and NaSO_4_ using DDC as a sulfur source. Higher adsorption capacities of MoS_2_/SH-MWCNT nanocomposite [Pb(ii) = 90.0 mg g^−1^ and Cd(ii) = 66.6 mg g^−1^] compared to O-MWCNTs [*Q*_m_, mg g^−1^ = 27.027 (Pb(ii)) and 24.4 (Cd(ii))] support the role of MoS_2_ in the adsorption efficiency.^17^ Li *et al.*, (2023) in this study, ferric nitrate modified carbon nanotube composites (FCNT) were prepared by isovolumetric impregnation using carbon nanotubes (CNTs) as the carrier and ferric nitrates the active agent. The batch experiments showed that FCNT could effectively oxidize As(iii) to As(v) and react with it to form stable iron arsenate precipitates. When the dosage of FCNT was 0.1 g L^−1^, pH value was 5–6, reaction temperature was 35 °C and reaction time was 2 h, the best arsenic removal effect could be achieved, and the removal rate of As(v) could reach 99.1%, which was always higher than 90% under acidic conditions. The adsorption results of FCNT were found to be consistent with Langmuir adsorption by static adsorption isotherm fitting, and the maximum adsorption capacity reached 118.3 mg g^−1^.^18^ Altaf, *et al.* (2021) in the current study, polydopamine carbon nanotubes (PD-CNTs) and polysulfone (PS) composite membranes were prepared. The potential application of PDCNs for heavy metal removal was studied for the removal of Pb^2+^, Cr^6+^, and Cd^2+^ from wastewater. The maximum removal efficiency of 96.1% was obtained for Cr^6+^ at 2.6 pH using a composite membrane containing 1.0% PD-CNTs. The removal efficiencies decreased by 64.1 and 73.4, respectively, by enhancing the pressure from 0.50 up to 0.85 MPa. Under the same circumstances, the percentages of Pb^2+^ removal at 0.49 bar by the PDCNS membranes containing 0.5% and 1.0% PD-CNT were 70 and 90.3, respectively, and decreased to 54.3 and 57.0, respectively, upon increasing the pressure to 0.85 MPa.^19^ Darvishi, *et al.*, (2023) recently, various emerging pollutants have been detected in water and wastewater, among which antibiotics can be considered a real threat to human life and environment because of their unique properties (bio-accumulation, bio-magnification, resistance, and bio-transformation). Studies have used the intra-particle diffusion, pseudo-first-order and pseudo-second-order models to model the adsorption kinetics, where in most of them, the pseudo-second-order model was in full agreement with the experimental data. Furthermore, this comprehensive review highlighted that the major mechanisms for the adsorption of antibiotics on coated-CNTs are π–π interaction, electrostatic interaction, hydrophobic interaction, and hydrogen bonding. Present study, the adsorption follows the Langmuir model, which represents monolayer adsorption.^20^ In this study, Multi-walled carbon nanotubes (MWCNTs) are highly favored in the field of nanotechnology because of their unique physical and chemical properties, and their high specific surface area. They also have a uniform particle size distribution, making them ideal for removing heavy metals like arsenic. To increase the adsorption capacity of the adsorbent, new functional groups were introduced onto the MWCNTs by creating MWCNTs-COOH. These new composite materials with higher porosity than MWCNTs show great promise as effective adsorbents for treating wastewater. Innovations in the article, after functionalization using potassium permanganate (KMnO_4_) and sulfuric acid (H_2_SO_4_), the adsorbent was magnetized. Carbon nanotubes with internal dimensions ranging from 30 to 50 nm and a length range of 10 to 20 μm, with a purity of 95%, were carefully chosen for the study, and surprisingly, no previous studies have explored this area. The absorption process is influenced by the initial pH of the solution: at high pH values, a decrease occurs in absorption efficiency. Changes in pH also had an impact on the efficiency of arsenic removal. At pH < 7, removal efficiency is relatively high at different concentrations of arsenic, reaching its maximum at pH = 6. At higher pH values, removal efficiency decreases relatively. Bankole *et al.* (2021) aimed to apply polyhydroxylbutyrate functionalized carbon nanotubes for arsenic removal at pH = 5.65, mixing time = 10 min, and adsorbent concentration = 20 mg L^−1^. They report a pseudo-second-order equation for the adsorption dynamics. In our study, at a pH of about 2 and mixing time of 30 minutes, *R*^2^ was equal to 1 based on pseudo-second-order kinetics, which was consistent with the findings of Bankole *et al.* (2021). The removal percentage of other heavy metals with this adsorbent in the study by Bankole *et al.* was 15.92% for Fe, 77.95% for Ni, 99.34% for Cd, 98.85% for Pb, 83.08% for Cu, 18.34% for Zn, 98.19% for Cr, and 99.95% for As.^21^ In recent years, a lot of research has been focused on methods to produce magnetic nanoparticles. Furthermore, the utilization of nanoparticles is continually expanding. The size, phase percentage, and shape of these nanoparticles are crucial depending on how they will be used. Magnetic nanoparticles are made through various methods and have many applications in different fields. The success of their use largely depends on how stable the nanoparticles are under different conditions. The most common method for preparing magnetic nanoparticles at the nanoscale is chemical co-precipitation. Khalafalla^22^ and his team prepared magnetic nanoparticles using this method, as shown in the TEM image (4). In this approach, a mixture of FeCl_2_ and FeCl_3_ in water is used to initiate the co-precipitation reaction according to the equation shown. The type, concentration, pH, temperature, and type of base significantly impact the size, efficiency, and magnetic properties of iron oxide nanoparticles. In the study by Li *et al.* (2021), a graphene oxide adsorbent was magnetized with Fe_3_O_4_, which increased the number of pores and enhanced adsorption capacity from 84.21 to 158.72 m^2^ g^−1^. However, our study involves the removal of arsenic with multi-walled magnetic carbon nanotubes (Fe_3_O_4_). Adsorbents doped with Fe_3_O_4_ are effective in removing pollutants from acidic mine drainage, especially the high levels of heavy metals released by the mining industry.^23^ According to Ambashta, R. D. *et al.* (2010), magnetic nanoadsorbents have better efficiency in removing metal ions such as Cr, Co, Ag, Cu, As(iii), and AS(v). These adsorbents are also used to remove organic, inorganic, radioactive, and algal pollutants, and reduce BOD, TN, and TP.^24^

## 2. Material and methods

### 2.1. Chemicals

A 1000 mg L^−1^ arsenic stock solution was prepared by dissolving 1.32 g of As_2_O_3_ in 1 liter of distilled water. Four grams of NaOH was used to increase the solubility of arsenic oxide (As_2_O_3_). HCl (0.1 M) and NaOH (0.1 M) were used to adjust the pH at the beginning of the adsorption test. All chemicals were purchased from Merck (Germany), including arsenic oxide (As_2_O_3_), iron chloride II (FeCl_2_·4H_2_O), iron chloride III (FeCl_3_·6H_2_O), hydrochloric acid (HCl), sodium hydroxide (NaOH), potassium permanganate (KMnO_4_), and sulfuric acid (H_2_SO_4_). Powdered carbon nanotubes were procured in the form of a commercial MWCNTs (95%) powder from US Research Nanomaterials, Inc.

### 2.2. Synthesis of MWCNTs

**Fig. 1:**
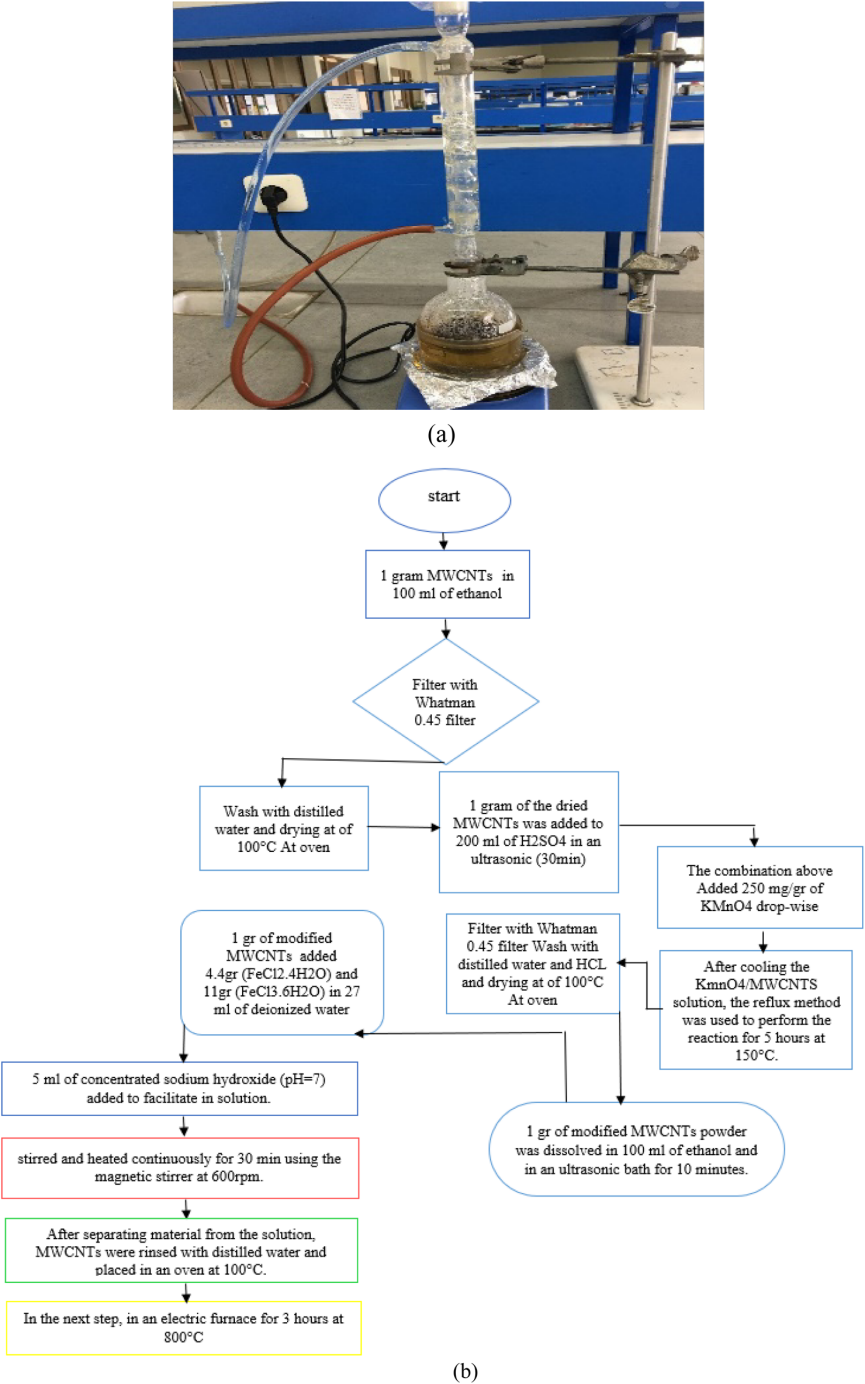
The reflux setup (a) flowchart for method steps and laboratory system used to manufacture (b).

Carbon nanotubes had 95% purity with an internal diameter of 30–50 nm and length of 10–20 μm. First, 1 gram of carbon nanotubes was added to 100 mL of 95% ethanol and dispersed using an ultrasonic device at room temperature for 1 hour. This helps to increase the total area of the adsorbent. The solution was then filtered with 0.45 Whatman filter paper followed by rinsing with distilled water and drying at of 100 °C to remove excess water. One gram of the dried carbon nanotubes was added to 200 mL of 0.5 M sulfuric acid (98%) and was placed in an ultrasonic device for 30 minutes. Next, the (MWCNTs/H_2_SO_4_) solution was combined with a 250 mg g^−1^ solution of KMnO_4_. The sulfuric acid solution was diluted with 100 cc of distilled water to prevent a rapid exothermic reaction (Fig. 1). After mixing carbon nanotube with H_2_SO_4_, it was added to KMnO_4_ drop-wise (in case of lack of the needed instrument, H_2_SO_4_ can be diluted with water (100 cc)). The resulting combination is capable of exploding immediately and simultaneously. After cooling the KmnO_4_/MWCNTS solution, the reflux method was used to perform the reaction for 5 hours at 150 °C. After separating the nanotubes from the solution with 0.45 Whatman filter paper and HCl (37%), the nanocomposite was rinsed with distilled water, followed by drying in an oven at 100 °C for 5 hours.

### 2.3. Synthesis of multi-walled carbon nanotubes magnetized with Fe_3_O_4_ nanoparticles

To make the magnetic carbon nanotubes, 1 g of modified carbon nanotube powder was dissolved in 100 mL of ethanol and placed in an ultrasonic bath for 10 minutes. Iron chloride II (FeCl_2_·4H_2_O) and iron chloride III (FeCl_3_·6H_2_O) were mixed at a low stirring speed with a molar ratio of 4.4 : 11 in 27 mL of deionized water; 5 mL of concentrated sodium hydroxide (pH = 7) was also added to facilitate the base substrate in solution. Using a heating magnetic stirrer, the solution was heated to 90 °C at 600 rpm. When the required temperature was achieved, the iron mixture was added dropwise to the prepared carbon nanotubes and stirred and heated continuously for 30 minutes using the magnetic stirrer at 600 rpm. After separating the synthesized material from the solution, carbon nanotubes were rinsed with distilled water and placed in an oven at 100 °C until completely dry. In the next step, the synthesized material was transferred to a heat-resistant container using a spatula and placed in a glass reactor (diameter: 50 mm, length: 100 mm) in an electric furnace for 3 hours at 800 °C.^25^ The adsorbent was separated using a 1.3 Tesla cylindrical magnet and stored inside a desiccator for later use.

### 2.4. Characterization

X-ray diffraction (XRD) was performed to determine the X-ray diffraction pattern of the adsorbent using a Nova 2000 machine (Quant Chrome). The morphology of adsorbent surface and the shape and size of Fe_3_O_4_ magnetic particles on carbon nanotubes were analyzed using field emission scanning electron microscopy (FESEM) with a FESEM Zelss Sigma 300 electron microscope. The phases present in the microstructure of the material were identified using energy-dispersive X-ray spectroscopy (EDS). The structural properties of the magnetized adsorbent were investigated using a transmission electron microscope (TEM, Philips EM208s). BET analysis was performed using the BELSORP-mini II machine to evaluate the adsorption of gas molecules on the surface of the solid. Thermogravimetric analysis and differential thermal analysis (TGA-DTA) were performed to assess changes in the physical and chemical behavior of the material under temperature change using a p600 device (USA).

A vibrating-sample magnetometer (VSM) was utilized to study the magnetic properties of the material. Finally, liquid chromatography-inductively coupled plasma mass spectrometry (ICP-MS) was used to detect arsenic (PerkinElmer, USA).

### 2.5. Adsorption test in the discontinuous system

This experimental study was performed at a laboratory scale. The parameters studied during the adsorption process include pH (5 levels), contact time (7 levels), amount of adsorbent (4 levels), and initial arsenic concentration (6 levels). The parameters and the number of levels for each were determined based on previous studies and the authors' past experiences. All samples were analyzed with three replications. Testing was performed in 50 mL Erlenmeyer flasks. Isotherms and adsorption kinetics under optimal conditions were investigated and the related parameters were calculated. The adsorption capacity of the synthesized adsorbent for arsenic and the adsorption rate were determined using eqn (1) and (2), respectively.^26^1
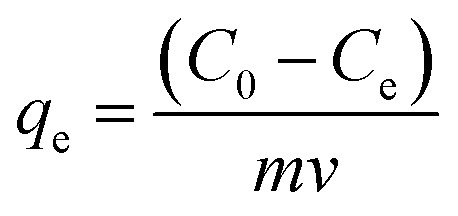
2
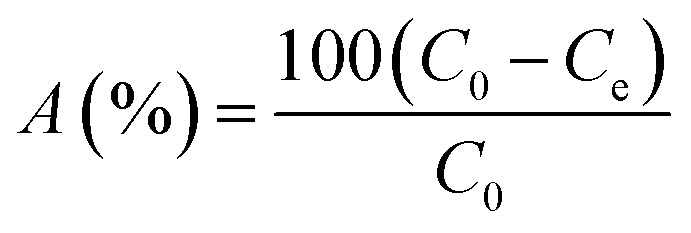
where *q*_e_ is the amount of arsenic per gram of adsorbent (mg g^−1^), *C*_0_ is the initial concentration of arsenic (mg L^−1^), *C*_e_ is the equilibrium concentration of arsenic after adsorption (mg L^−1^), *v* is the volume of solution (L), and *m* is absorbent mass (g).

### 2.6. Arsenic adsorption parameters

In order to optimize the process, first the effect of pH (between 2 and 10) was investigated on the adsorption of arsenic and its optimal value was determined. pH was adjusted using 0.1 M HCl and 0.1 M NaOH solutions. After determining the optimal pH, the effect of contact time (15–240 minutes) was determined at the optimal pH. These two parameters were determined using an initial arsenic concentration of 6 mg L^−1^ and 0.02 g of adsorbent. Next, the effects of the initial concentration of arsenic (2–12 mg L^−1^) and different amounts of adsorbent (0.02–0.1 g L^−1^ range) were investigated. Finally, isotherm equations and adsorption kinetics were formulated in order to determine adsorption capacity, reaction rate, and the model for the adsorption process.

#### 2.6.1. Adsorption isotherms

Isotherms are an important parameter in the design of adsorption systems and describe the relationship between the concentration of the adsorbate and the adsorption capacity of an adsorbent. Models and equations for equilibrium adsorption isotherms are used to describe the adsorption proprieties of the adsorbent. In this study, Langmuir and Freundlich equilibrium isotherm models were used. The Langmuir isotherm model is based on the adsorption of a uniform (homogeneous) layer of adsorbate on the adsorbent with the same energy on all surfaces, whereas the empirical Freundlich isotherm equation is based on multilayer heterogeneous adsorption of the adsorbate on adsorbent. The equations for these isotherms are respectively presented in eqn (3) and (4).^27^

Langmuir equation:3
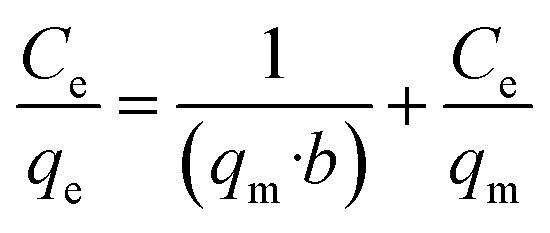
where *q*_e_ is the amount of arsenic adsorbed under equilibrium (mg g^−1^), *C*_e_ the concentration of the adsorbate under equilibrium (mg L^−1^), *q*_m_ is the maximum adsorption capacity of the adsorbent (mg g^−1^), and *b* is the Langmuir coefficient (the equilibrium constant for dispersion of the metal ion between the solid and liquid phases).

Freundlich equation:4
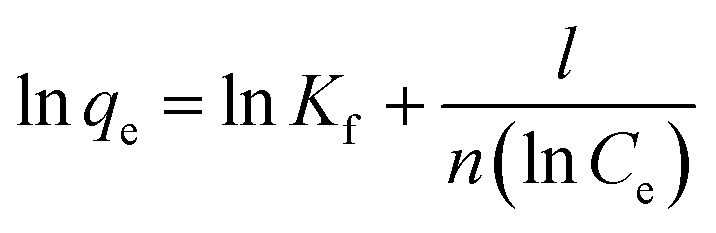
where *n* and *K*_f_ are the Freundlich constants; *n* indicates adsorption intensity and *K*_f_ represents the adsorption capacity of the adsorbent at constant concentration (mg g^−1^ (mg L^−1/*n*^)). In this model, *n* < 1 indicates weak adsorption, and 1 < *n* < 2, and 2 < *n* < 10 indicate intermediate and ideal adsorption, respectively. *n* is calculated as the slope of ln *q*_e_ against ln *C*_e_, and *K*_f_ as its *y* intercept.

#### 2.6.2. Absorption kinetics

Kinetic equations are used to investigate factors affecting the reaction rate. In the present study, pseudo-first-order and pseudo-second-order kinetic models were used to model arsenic adsorption on Fe_3_O_4_-f/MWCNTs. The pseudo-first- and second-order linear kinetic relations are expressed in eqn (5) and (6), respectively.^28,29^

Pseudo-first-order kinetics assumes that infiltration occurs from within a layer according to the solid's permeability and that changes in adsorption over time are proportional to the number of unoccupied sites on the nanoparticle's surface.

5 ln(*q*_e_ − *q*_*t*_) = ln *q*_e_ − *k*_1_*t*

where *q*_e_ is the amount of arsenic adsorbed under equilibrium (mg g^−1^), *q*_*t*_ is the amount of arsenic absorbed at time *t*, and *k*_1_ is the rate constant of the first-order equilibrium (min^−1^). *k*_1_ is the slope of the line created by plotting ln(*q*_e_ − *q*_*t*_) against *t*. The pseudo-second-order kinetic model assumes that chemical adsorption is the rate-limiting step and takes place in the solid phase. In this model, the rate of occupation of adsorption sites is proportional to the square of the number of unoccupied sites.6
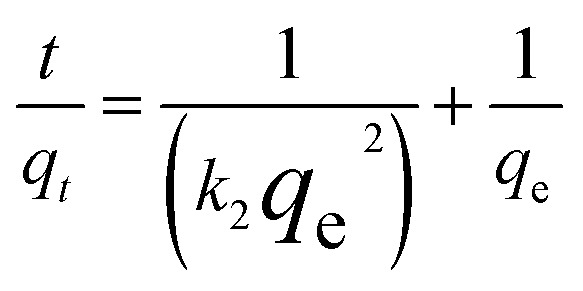
where *k*_2_ is the second-order equilibrium constant (mg g^−1^ min^−1^). *k*_2_ and *q*_e_ are obtained by calculating the slope and *y* intercept of the graph. Slope and width are obtained from the origin of the graph.

## 3. Results and discussions

### 3.1. Morphological properties

**Fig. 2:**
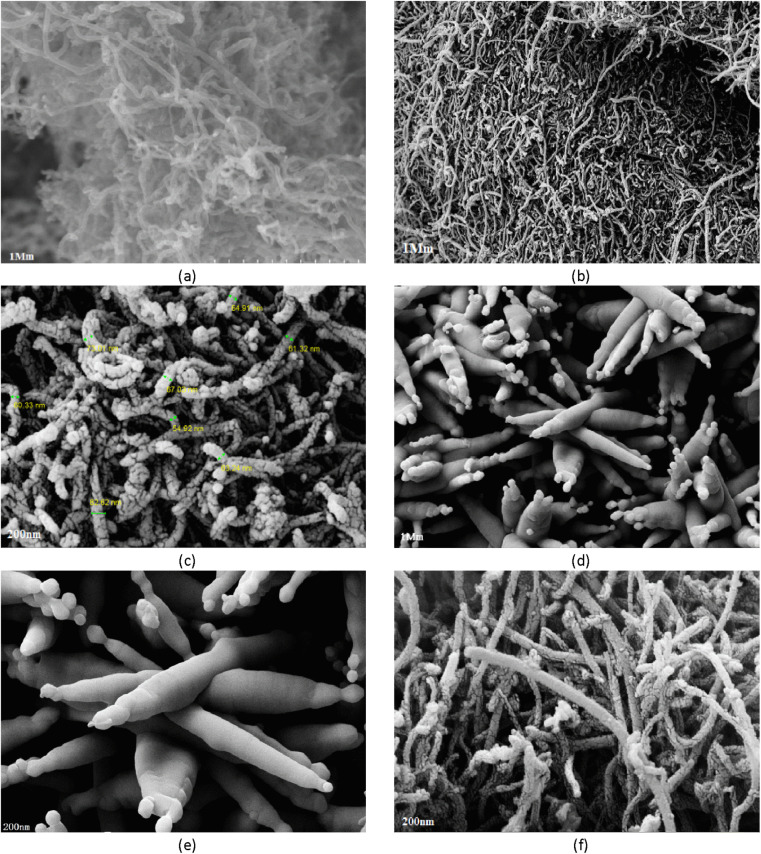
FESEM image of MWCNTs (a); Fe_3_O_4_-f/MWCNTs (b and c); Fe_3_O_4_ (d and e); Fe_3_O_4_-MWCNTs-As (f). The scale bar respectively represents (a and b) 1 μm, (c) 200 nm, (d) 1 μm, and (e and f) 200 nm.

Morphological properties of the synthesized adsorbent were studied using FESEM-EDX. Fig. 2 shows the surface properties of the CNTs as captured by FESEM at 10 keV with a magnification of 10–5 × 10^5^×. The figures show pores of different sizes on the surface of CNTs and magnetized CNTs. The average size of Fe_3_O_4_-f/MWCNTs particles was 82.62 nm. Compared to MWCNTs, Fe_3_O_4_-f/MWCNTs show a greater size distribution. The adsorbent surface is not uniform and many pores are observed on its surface.

### 3.2. Energy dispersive X-ray spectroscopy (EDS)

**Fig. 3:**
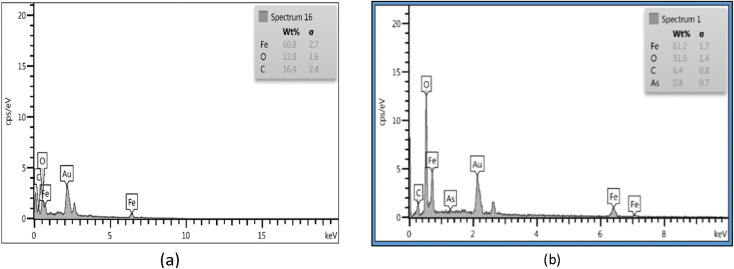
Results of EDS for Fe_3_O_4_-f/MWCNTs (a); Fe_3_O_4_-MWCNTs-As (b).

EDS showed that the chemical composition of Fe_3_O_4_-f/MWCNTs is 16.4% carbon, 22.8% oxygen, and 60.8% iron. The figures for Fe_3_O_4_-MWCNTs-As were 31.6% oxygen, 61.2% iron, 6.4% carbon, and 0.8% arsenic (Fig. 3).

### 3.3. Structural properties

**Fig. 4:**
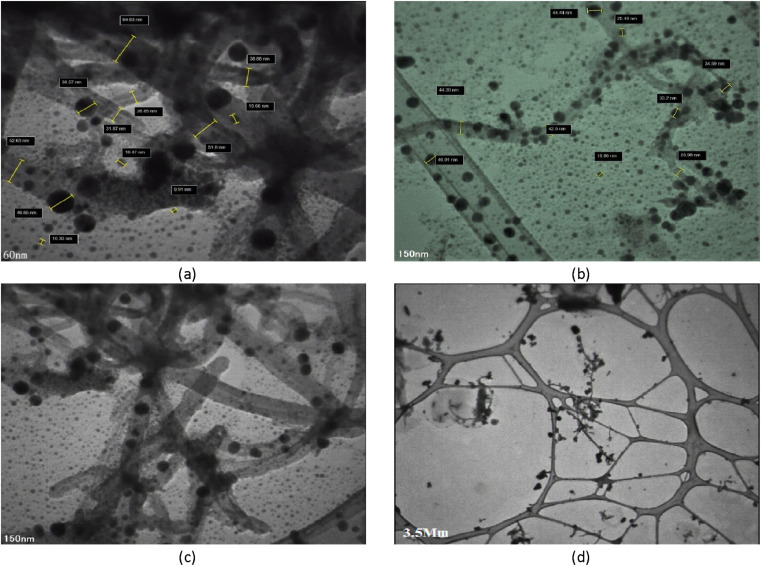
TEM Image of Fe_3_O_4_-f/MWCNTs, scale bar: (a) 60 nm, (b and c) 150 nm, (d) 3.5 μm.

The structural properties of the adsorbent were studied using TEM (Fig. 4). Comparing FESEM and TEM images reveals a great degree of agreement, indicating that magnetic particles were successfully synthesized and deposited on CNTs. The iron oxide nanoparticles on CNTs are intertwined and form an almost non-homogenous structure composed of many nanocrystals; the small particles have a size of 8.6 nm and the large particles have a size of 76.79 nm.

### 3.4. FT-IR spectroscopic analysis

**Fig. 5:**
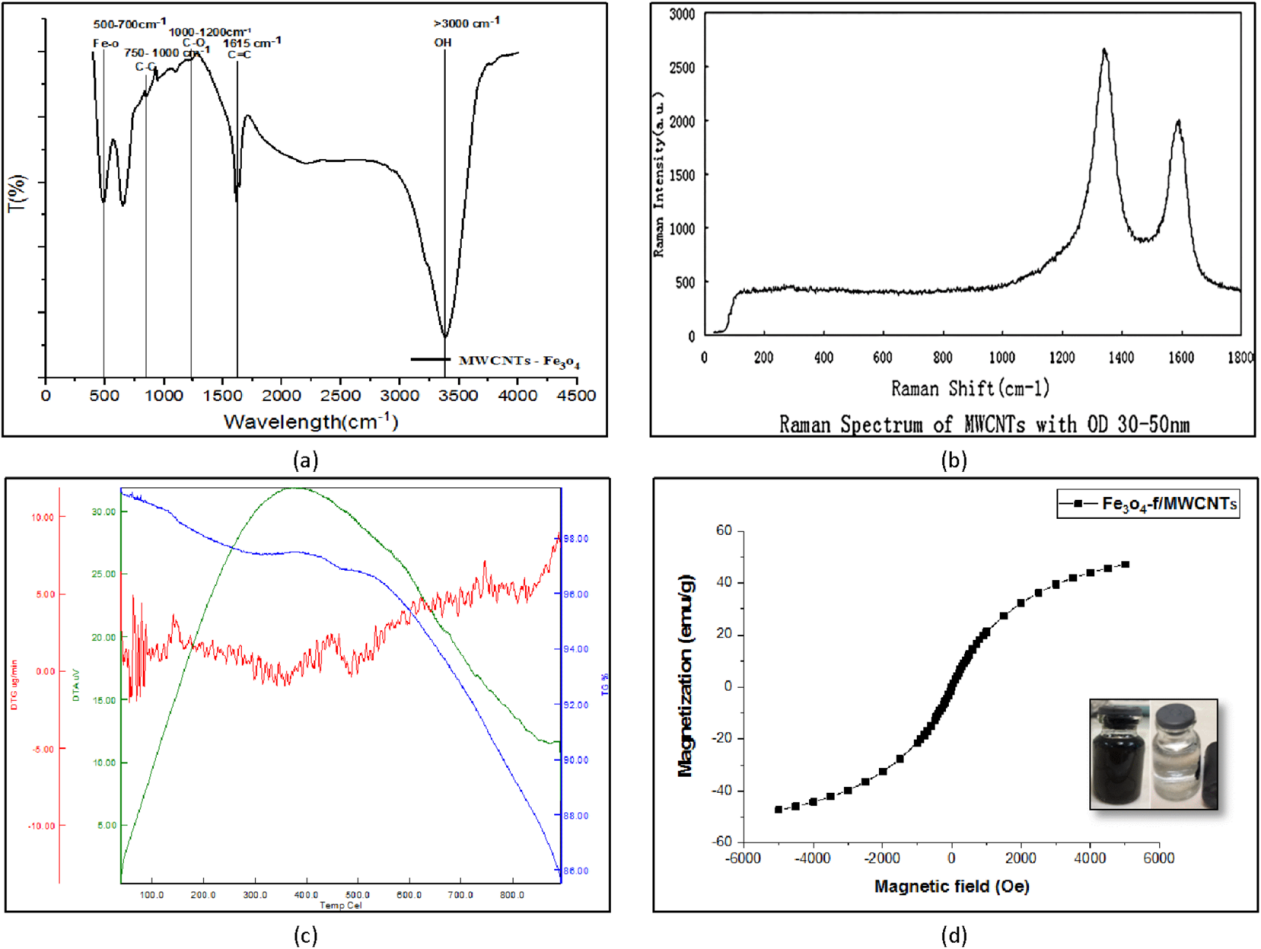
FT-IR spectroscopy of Fe_3_O_4_-f/MWCNTs (a) and Raman spectrum of MWCNTs (b), TGA-DTG patterns of Fe_3_O_4_/f-MWCNTs(c), VSM for Fe_3_O_4_-f/MWCNTs (d).

Fourier-transform infrared (FT-IR) spectroscopy is used to measure and determine the structure of chemical species in a sample. The peaks in the 3000–3500 cm^−1^ region correspond to O–H stretching in ethers. The peaks at 1615 cm^−1^, 1000–1200 cm^−1^, 750–1000 cm^−1^, 500–700 cm^−1^ are respectively associated with C=O, C=C, C–C, and Fe–O–Fe bonds (Fig. 5a).

Fig. 5b shows the results of Raman spectroscopy for CNTs. This technique is a suitable method for the detection of carbonaceous materials based on differentiating sp, sp^3^, and turbotrain sp^2^ carbon atoms. The peak at 1330 cm^−1^ corresponds to carbon bonds (G band) and the peak at 1580 cm^−1^ indicates structural defects (D band). This phenomenon indicates that some of the carbon bonds have been broken down by acids, forming carboxylic (–COOH) and hydroxyl (–OH) functional groups (Fig. 5b).

### 3.5. Thermal properties

TGA-DTA was used to evaluate the thermal properties of the synthesized nanocomposite. TGA showed that the CNTs have a relatively stable structure which decomposes at 100 °C. At this temperature, CNTs lose approximately 5% of the water adsorbed on their surface. At 500 °C about 95% of the base structure remains stable. The structure remained stable up to about 800 °C. The CNTs lose weight at 334.515, 470.304, and 799.529 °C, leading to a final 15% decomposition. Fig. 5c shows the results of TGA-DTA analysis for the nano-adsorbent.

### 3.6. VSM test

One of the common methods for investigating the magnetic properties of materials is the VSM test. Fig. 5d shows the hysteresis curve for magnetized carbon nanotubes at room temperature in +6000 to −6000 oersted fields. The figure clearly shows that hysteresis is minimal and field generation and remanence are very small. According to the research done by Myrovali, *et al.*, 2016 this magnetic hysteresis curve shows a behavior similar to that of superparamagnetic materials in terms of changes in magnetism. In other words, a hysteresis loop is not observed in this curve. It can be seen in Fig. 5 that the magnetization of the magnetized CNTs is 47.32 emu g^−1^.^30^

### 3.7. X-ray diffraction (XRD)

**Fig. 6:**
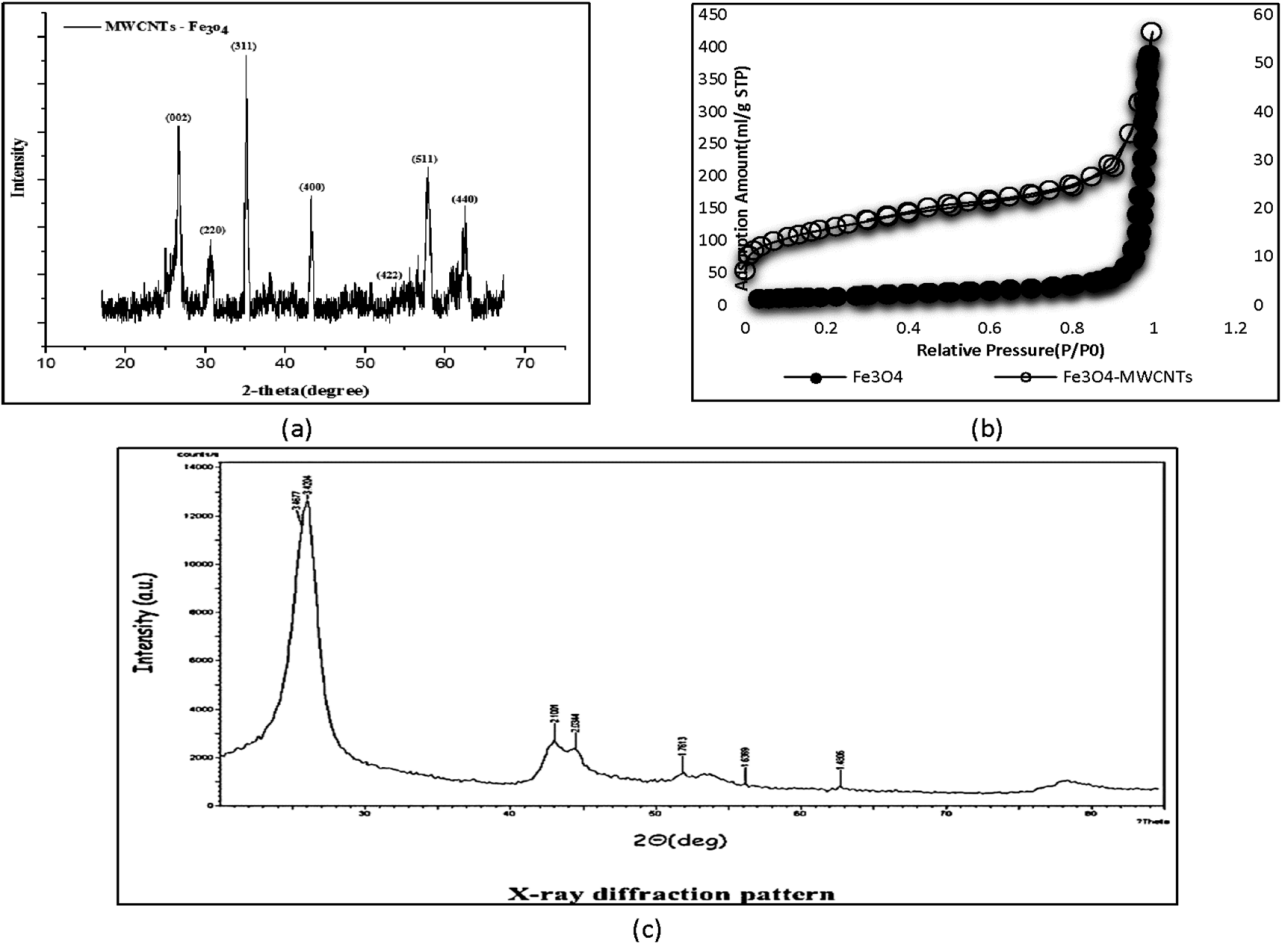
XRD patterns of Fe_3_O_4_-f/MWCNTs (a), N_2_ adsorption isotherm at 77 K for Fe_3_O_4_-MWCNTs and Fe_3_O_4_ (b) and X-ray MWCNTs (c).

An external magnet can be used to assess the magnetic properties of the synthesized adsorbent and separate the material. The diffraction pattern for Fe_3_O_4_ is presented at 2*θ* = 15°–70° in Fig. 6a. The peaks at 30.2°, 35.3°, 43.26°, 54.45°, 57.04°, and 63.52° indicate the presence and deposition of iron (Fe_3_O_4_) in the structure of CNTs. The sharp and distinct graphite peak at 2*θ* = 25° is evidence of carbon formation. The X-ray diffraction spectrum of CNTs is shown in Fig. 6c. The two maxima at 3.42 and 3.18 correspond to the structure of graphene and CNTs.

### 3.8. Specific surface and pore size

One of the most important methods for measuring the specific surface area of porous materials is the BET method, which is based on the adsorption of specific molecular species from the gaseous phase on the material's surface. The IUPAC classification describes the structure of a porous medium according to the average dimension of pores, and places pores in three classes: micropores (average pore diameter < 2 nm), mesopores (2 < average pore diameter < 50), and macropores (average pore diameter > 50). The specific surface areas of Fe_3_O_4_-MWCNTs and Fe_3_O_4_ were 549 m^2^ g^−1^ and 52.05 m^2^ g^−1^, respectively (Table 1). The surface area of magnetic adsorbent increased after saturation. Saturation of pores with magnetic particles usually reduces the base surface (Fig. 6b).

**Table 1:** Surface area, total pore volume, and pore diameter of Fe_3_O_4_-MWCNTs and Fe_3_O_4_

Sample	Surface area (m^2^ g^−1^)	Total pore volume (cm^3^ g^−1^)	Average pore diameter (nm)
Fe_3_O_4_-MWCNTs	549	7.98	0.98
Fe_3_O_4_	52.05	0.5938	5.81

## 4. As adsorption

### 4.1. The effect of pH

**Fig. 7:**
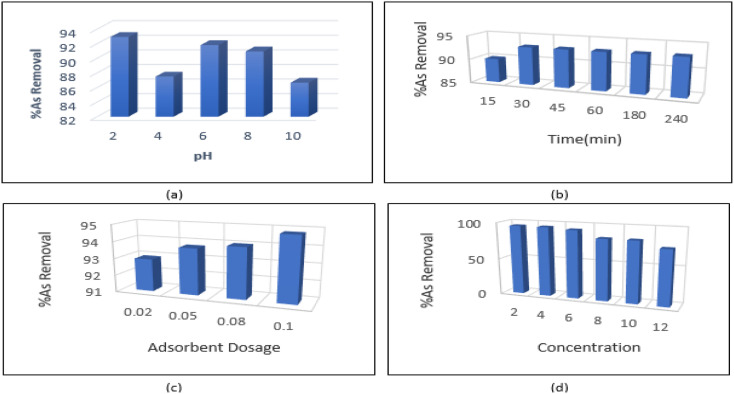
(a) As(iii) removal rate at different pH, (absorbent dose: 0.02 g L^−1^, arsenic concentration: 6 mg L^−1^), (b) effect of time on arsenic removal efficiency (pH = 2, arsenic concentration = 6 mg L^−1^) (c) effect of amount of adsorbent on removal efficiency (pH = 2, arsenic concentration = 6 mg L^−1^), (d) effect of arsenic concentration on arsenic removal efficiency (pH = 2, contact time = 30 min, adsorbent concentration = 2 mg L^−1^).

As shown in Fig. 7a, the maximum amount of arsenic was adsorbed at pH = 2. The pH of the solution affects the surface charge of the nanoparticles. The distribution of functional groups such as carboxyl and hydroxyl can be calculated by acid–base titration. pH has a significant effect on arsenic absorption; at acidic pHs the adsorption efficiency of arsenic on the adsorbent is higher than alkaline conditions. Therefore, a strong attraction force is created between the surface groups and the pollutant, which results in increased adsorption. However, at pHs above the isoelectric point of CNTs, the negatively-charged adsorbent surface becomes electrostatically repulsive and ultimately reduces the adsorption rate. In an alkaline environment, the activity of carbonyl, carboxyl and hydroxyl groups which are responsible for pollutant adsorption is increased due to the presence of OH^−^ ions. As a result, the adsorption rate decreases due to lowered surface adsorption.

### 4.2. The effect of contact time

We studied the effect of contact time (ranging from 15–240 minutes) on adsorption and found that absorption rate increased rapidly in the first 30 minutes. From 30 minutes to 240 minutes, the absorption curve showed a gentler slope. The mixture reached equilibrium in about 30 minutes. This is due to the high effective surface area of carbon nanotubes, which causes the diffusion of pollutant ions from the solution to active sites on the surface CNTs. The rapid increase in adsorption capacity in the early stages of the process may be due to the high availability of saturated sites to arsenic ions on the surface of CNTs. As the process continues, access to surface sites is reduced, leading to equilibrium sometime after the beginning of the process. After equilibrium is reached, changes in adsorption may be very small, or in some cases, adsorption may occur from the surface of the adsorbent into the solution. Maximum adsorption efficiency was achieved in 30 minutes and therefore 30 minutes was chosen as the equilibrium time to continue the experiments (Fig. 7b).

### 4.3. Effect of amount of adsorbent

In order to determine the optimal amount of adsorbent, tests were conducted using 0.02–0.1 g L^−1^ of the synthesized material. The effect of amount of adsorbent on adsorption efficiency is shown in Fig. 7c. As the number of adsorbent particles and the sites available for adsorption increase, adsorption efficiency also increases. The smallest amount of adsorbent (0.02 g L^−1^) was selected to investigate the arsenic adsorption process (Fig. 7c).

### 4.4. Effect of pollutant concentration

At this stage of the experiment, the most suitable concentration of metal to be removed by the adsorbent was determined under the optimal conditions (pH = 2, contact time = 30 min, adsorbent concentration = 2 mg L^−1^). According Fig. 7d, the optimal absorption rate occurred at a concentration of 6 mg L^−1^. At higher As concentrations, adsorption showed a decreasing trend.

### 4.5. Adsorption isotherm

**Fig. 8:**
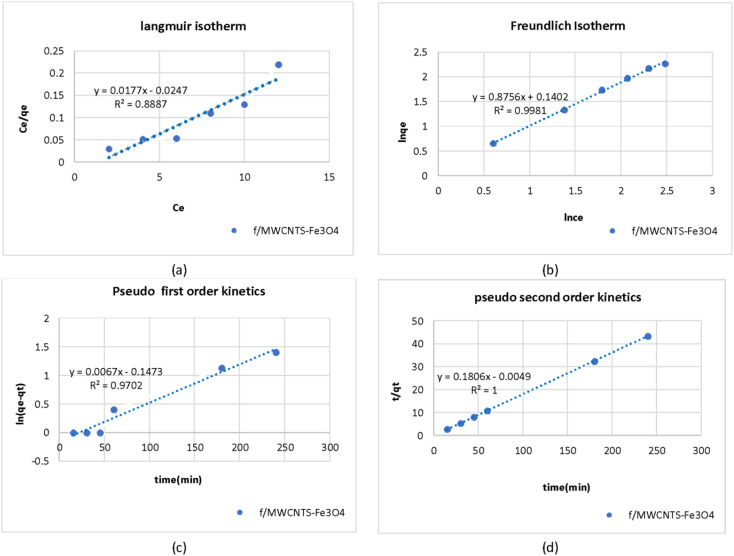
Adsorption isotherms for adsorption of As(iii) on Fe_3_O_4_-f/MWCNTs using linearized Langmuir and Freundlich isotherms; pseudo-first-order kinetics and pseudo-second-order kinetics for adsorption of AS(iii) by Fe_3_O_4_-f/MWCNTs.

Fig. 8a and b shows the values of the parameters calculated from the Langmuir and Freundlich isotherms for adsorption of As on Fe_3_O_4_-f/MWCNTs in an aqueous solution. Absorption capacity (*q*_m_) was 0.26 mg g^−1^. The coefficient of determination (*R*^2^) clearly shows that the arsenic adsorption process by Fe_3_O_4_-f/MWCNTs more closely follows the Freundlich equilibrium isotherm.

Table 2 presents the isotherm parameters for Langmuir and Freundlich isotherms as well as the results of regression analysis. A comparison of the coefficients of determination (*R*^2^ = 0.9981 *vs. R*^2^ = 7032) reveals that the adsorption process follows the Freundlich isotherm model. This indicates that the adsorbate is adsorbed in a multilayered and heterogeneous manner on the adsorbent. Also, the value of RL in Langmuir model was between 0 and 1 and the Freundlich constant (1/*n*) was smaller than one, indicating favorable adsorption of As on the magnetized CNTs.

**Table 2:** Parameters of Langmuir and Freundlich isotherm models

Langmuir model			Freundlich model		
*B* (L mg^−1^)	*q*_m_ (mg g^−1^)	*R* ^2^	*K*_f_ (mg g^−1^)	1/*n*	*R* ^2^
0.35	0.26	0.8887	1.38	0.87	0.9981

The Langmuir isotherm can be interpreted using a separation factor (RL) calculated as 1/(1 + *kLC*_0_). RL > 1 represents unfavorable adsorption, RL = 0 represents irreversible adsorption, and RL = 1 represents linear adsorption. Favorable adsorption is achieved when 0 < RL < 1. Fig. 8 presents adsorption capacity and the value of the coefficient of determination (*R*^2^) based on the Langmuir model for adsorption of As on Fe_3_O_4_-f/MWCNTs. The effects of the initial concentration of As on adsorption can also be seen in the figure.

### 4.6. Pollutant adsorption kinetics

Table 3 presents the values calculated for the pseudo-first- and second-order kinetic parameters for arsenic adsorption on Fe_3_O_4_-f/MWCNTs. Comparing the coefficients of determination (*R*^2^) for the two models shows that the process follows a pseudo-second-order kinetic model. Fig. 8c and d shows the linear models for the adsorption process, indicating that the amount of adsorbed arsenic on CNTs follows a pseudo-second-order model. In addition, there is very little difference between the calculated *q*_c_ and the observed *q*_e_, which indicates that arsenic adsorption follows a chemical adsorption mechanism dominated by electron sharing between arsenic and the active sites on the surface of magnetized CNTs (Fig. 8).

**Table 3:** Parameters for kinetic equations

Pseudo-first-order model			Pseudo-second-order model		
*q*_m_ (mg g^−1^)	*K*_1_	*R* ^2^	*q*_m_ (mg g^−1^)	*K*_2_	*R* ^2^
1.158	0.0067	0.9702	5.98	69.97	1

## 5. Conclusion

The optimal conditions for arsenic removal by the synthesized Fe_3_O_4_-bearing CNTs were evaluated under the effect of pH (2–10), contact time (15–240 minutes), amount of adsorbent (0.02–0.1 g), and initial concentrations of arsenic (2–12 mg). The optimal conditions found in this study can maximize adsorption efficiency and provide a better understanding of the function of different parameters and their interactions. For example, the results showed that at pH values below 6, the speed and amount of adsorbent significantly change removal efficiency.

Magnetic adsorbents are easily and quickly separated from aqueous media and have better performance compared to conventional micro-adsorbents due to their high specific surface area and low dispersion resistance. This study showed that magnetic carbon nanotubes have great potential to remove arsenic pollution. Therefore, this material can be used to remove such pollutants from aquatic environments.

## Conflicts of interest

The authors declare no conflict of interest.

## Supplementary Material
